# Development of Novel Adenoviral Vectors to Overcome Challenges Observed With HAdV-5–based Constructs

**DOI:** 10.1038/mt.2015.194

**Published:** 2015-11-24

**Authors:** Julio Alonso-Padilla, Tibor Papp, Győző L Kaján, Mária Benkő, Menzo Havenga, Angelique Lemckert, Balázs Harrach, Andrew H Baker

**Affiliations:** 1 Institute of Cardiovascular and Medical Sciences, College of Medicine, Veterinary and Life Sciences, University of Glasgow, Glasgow, UK; 2 Institute for Veterinary Medical Research, Centre for Agricultural Research, Hungarian Academy of Sciences, Budapest, Hungary; 3 Batavia Biosciences B.V., Leiden, The Netherlands; 4 Current address: Centre for Cardiovascular Sciences, Queen's Medical Research Institute, University of Edinburgh, Edinburgh, UK

## Abstract

Recombinant vectors based on human adenovirus serotype 5 (HAdV-5) have been extensively studied in preclinical models and clinical trials over the past two decades. However, the thorough understanding of the HAdV-5 interaction with human subjects has uncovered major concerns about its product applicability. High vector-associated toxicity and widespread preexisting immunity have been shown to significantly impede the effectiveness of HAdV-5–mediated gene transfer. It is therefore that the in-depth knowledge attained working on HAdV-5 is currently being used to develop alternative vectors. Here, we provide a comprehensive overview of data obtained in recent years disqualifying the HAdV-5 vector for systemic gene delivery as well as novel strategies being pursued to overcome the limitations observed with particular emphasis on the ongoing vectorization efforts to obtain vectors based on alternative serotypes.

## Introduction

**Figure 1:**
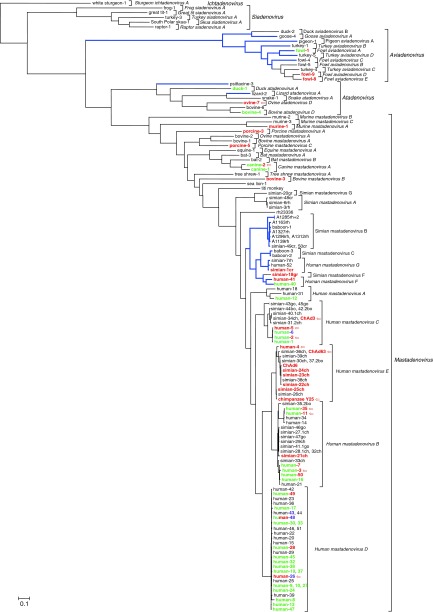
**The *Adenoviridae* diversity tree**. Maximum likelihood analysis of the full DNA-dependent DNA polymerase amino acid sequences to show the evolutionary distance of the fully sequenced adenovirus serotypes and certain not serotyped strains. Model selection by ProtTest proposed LG+I+G. User tree gained by distance matrix analysis (ProtDist by JTT, Fitch followed by global rearrangement). The PhyML calculated tree is visualized by Mega6. Nonrooted calculation. For visualization of the supposed evolutionary history, the fish adenovirus (AdV; white sturgeon AdV-1) was applied as outgroup. Vectorized types/strains (if published) are shown by red and bold letters. (Porcine AdV-4 and fowl AdV-10 are not shown on the tree as their DNA polymerase genes have not been published. Neither are shown rhesus AdV-51 to -53 as their DNA polymerase sequences in GenBank are shorter than those of other adenoviruses most probably due to not recognizing their spliced nature). Vectors that have reached human clinical trials are designated by a red arrow. The HAdV-5 recombinants engineered with fibres of other AdV types are shown by green bold letters. The several human adenoviruses that have been both vectorized and their fibers pseudotyped on human adenovirus 5 are shown with their name in green and the number in red. When the hexon hypervariable regions were pseudotyped onto HAdV-5, the serotype number is shown by lilac letters. Branches of AdVs that have two fiber genes are shown by blue and thicker lines. Official species are shown in italics; proposed but not yet accepted species are in normal letters. Genera are shown in italics and bold. The scale bar shows the evolutionary distance of 0.1 aa substitution per position. The word of “adenovirus” is removed from the type and strain names. Abbreviated names after the type numbers show the hosts of the simian adenoviruses; bo: bonobo, ch: chimpanzee, cr: crab eating macaque; go: gorilla; gr: grivet; rh: rhesus macaque.

Adenoviruses (AdVs; family *Adenoviridae*) are medium size, nonenveloped DNA viruses (70–90 nm in diameter).^1^ They are classified in five genera with all human AdV (HAdV) serotypes belonging to the genus *Mastadenovirus* (**Figure 1**).^2^ HAdVs are further grouped within species *Human mastadenovirus A* to *G* (HAdV-A to G) based on their phylogeny, genome organization, G+C content, hemagglutination pattern, and other biological properties. At present, 56 distinct serotypes belonging to HAdV-A to G have been described. Serotype-dependent, HAdV infections are tropic to the eye, respiratory system, kidney, or gastrointestinal tract. Although HAdV infection poses a risk for immune-compromised individuals, infections are mostly subclinical in immunocompetent subjects.^3^

The best studied member of the HAdV species is serotype 5 (HAdV-5, species HAdV-C). Structural studies demonstrated that the HAdV-5 particle has an icosahedral capsid (~90 nm in diameter) that protects a double-stranded linear single DNA genome ~35 kb long.^4,5^ The capsid predominantly contains three proteins called hexon, penton base, and fiber which interact directly and are also held together by a defined number of so-called cement proteins.^6,7^ The hexon protein is the most abundant capsid protein and contains the hypervariable regions (HVRs) which are serotype-specific protein sequences and hence are considered major immune determinants.^8^ At each of the 12 icosahedron vertices, 5 penton polypeptides form a base (penton base) from which a trimeric fiber protein protrudes away. The fiber protein is known to be the main determinant of serotype tropism.^4,5^ For instance, for HAdV-5, it has been shown that the cellular coxsackievirus and adenovirus receptor (CAR), a tight junction protein, acts as its primary receptor whereby the HAdV-5 fiber protein binds CAR directly.^9^ It has been further shown that HAdV-5 virus internalization, upon binding to CAR, is promoted by the RGD protein motif present in the penton base by directly binding to cellular α_v_β_5_ integrins, a process that further involves clathrin-coated vesicles and dynamin-dependent endocytosis.^10,11^ Studies with other HAdV serotypes have identified that receptor molecules other than CAR can be utilized, like the cellular CD46 protein or desmoglein-2 by HAdV-B species, as well as sialic acid moieties of relevance to members of the HAdV-D species.^12^ Upon cell entry, the virus is located in endosomes and endosomal membrane rupture, mediated by the viral pVI, liberates semi-uncoated viral particles into the cell cytoplasm,^13^ which are then dynein trafficked to the nucleus.^11^

HAdV-5 infects many cell types, including low-replicative or quiescent cell populations and professional antigen-presenting cells. Owing to decades of intensive research, the HAdV-5 genome is now easy to engineer, yielding stable recombinant replication-deficient HAdV-5 particles with large foreign DNA cloning capacity. The virus genome remains episomal summoning a safer profile in comparison to many other viral vectors. Moreover, HAdV-5 vectors can be produced on an industrial scale under good manufacturing procedures achieving titers of up to 10^13^ replication-deficient virus particles per ml (VP/ml). All these attributes make HAdV-5 vectors the most preferred vector type used to date in vaccine, cancer, and gene therapy trials,^14,15^ and first in man products based on HAdV-5 have been approved.^16^ However, two decades of intensive research have also highlighted certain challenges associated with the use of HAdV-5 vectors that limit their clinical application. These include both a high innate immune toxicity profile associated with a marked liver tropism when HAdV-5 vectors are delivered intravenously (i.v.), and a worldwide high preexisting adaptive immunity (PEI) against HAdV-5 in man, observed also for many other common HAdV serotypes. These biological findings and the subsequent disqualification of HAdV-5 vectors for certain product indications is discussed. Also, ongoing research to find alternatives to HAdV-5 vectors usage is described, with special attention given to the discovery and vectorization of novel AdV types isolated from human and nonhuman tissues.

## Challenges with the Development of HADV-5–Based Medicinal Products

### Innate immunity-associated toxicity in response to HAdV-5 delivery

A high i.v. dose of vector (>10^13^ VP) has been shown to overwhelm the innate immune mediators leading to a systemic cytokine shock which eventually resulted in the death of a patient enrolled in a clinical gene therapy trial.^17^ It was argued that administration of such huge systemic doses was needed to surpass the HAdV-5 vector-sequestering pharmacological “sink” in human liver (see next section). Although other routes or *ex vivo* transgene delivery have been shown to be plausible approaches in some HAdV-5 applications,^18,19,20^ treatment of cardiovascular diseases or disseminated tumors requires the vectors to be delivered systemically.^21^ Moreover, vector bloodstream injection provides a more straightforward product application approach than, *e.g.*, surgically invasive delivery methods.

A major disadvantage for the vectors' interaction with host immunity is imposed by constraints in their genetic design, which in order to make them biologically safer (replication-deficient) and provide room for larger foreign DNA inserts, has crippled them from their inherent immune-evasive countermeasures (encoded by proteins transcribed from the viral E1, E3, and E4 regions).^22^ For instance, a HAdV-5 vector that still expressed genes located in the E3 region demonstrated prolonged transgene expression as compared to its E3-deleted counterpart upon i.v. injection in rats, thus demonstrating the ability of the HAdV-5-E3^+^ vector to escape immune eradication.^23^ Next to these genetic changes and their impact on HAdV-5 *in vivo* interaction with the immune system, it has also been described that expression of a foreign transgene can limit the survival of the HAdV-5 vector *in vivo*, although this clearly will be a challenge for the use of AdVs vectors in general. Here, the use of less toxic regulatory sequences should as well be considered.^24^

The HAdV-5 vector capsid proteins, dsDNA, and VA-RNAs have been shown to trigger innate host immune responses.^25,26^ From the first cell attachment event, through their endosomal trafficking, along their cytosolic presence, and final delivery of the viral genome into the cellular nucleus, HAdV-5 is exposed to cell molecular sensors (“pathogen recognition receptors”). Cell surface-located toll-like receptor 2 (and endosomal membrane-located toll-like receptor 9 recognize and respond to HAdV-5 capsid components.^27,28^ Nucleotide-binding oligomerization domain-like receptors were found to be involved in the recognition of HAdV-5 dsDNA patterns,^22^ and the cytosolic retinoic acid-inducible gene I was described to induce type I interferons in response to HAdV-5–derived VA-RNAs.^29^ Activation of pathogen recognition receptors leads to the setting of a cellular antiviral state involving the secretion of proinflammatory cytokines (TNFα, IL6, IL12, IFNγ, IL1α, and IL1β) and chemokines (RANTES, MCP-1, KC, MIP-1α, MIP1β, and IP10).^22^ Notably, innate immune responses to AdV vectors have been shown to be dose dependent for HAdV-5, but more importantly, it has also been demonstrated that the host innate immune response can be strikingly different in response to different HAdVs, or AdVs in general for that matter, spurring the search for other AdVs which are less prone to trigger a systemic cytokine storm upon *in vivo* administration.

### HAdV-5–associated hepatotoxicity

Whereas seemingly promising for hepatic gene therapy, liver sequestering of systemically delivered HAdV-5 vectors is a major problem when the vector needs to express the foreign transgene in other tissues. High doses (10^12^–10^13^ VP) of HAdV-5 have been administered in an attempt to counterbalance the liver sequestering, thereby risking hepatic injury and inflammatory shock syndrome as HAdV-5-damaged liver macrophages are a major source of proinflammatory cytokines.^30^ Before reaching the liver, HAdV-5 vectors interact with multiple blood components, *i.e.*, erythrocytes, thrombocytes, and circulatory proteins like immunoglobulins, complement system, and blood coagulation factors.^30^ For instance, HAdV-5 binding to erythrocytes was reported to take place directly via CAR and complement receptor 1.^31,32^ As the most abundant blood cell type, erythrocyte interaction with HAdV-5 is of high pharmacological relevance. Hence, generally accepted mouse and nonhuman primate models may not accurately depict HAdV-5 vector biodistribution since their erythrocytes do not express CAR.^32,33^ Naturally occurring immunoglobulins have been described to influence HAdV-5 vector pharmacology too, gating vectors' clearance by the liver,^34,35,36^ a process that was shown to be favored by complement system factors.^34^

Of great relevance in HAdV-5 vector liver tropism, a high-affinity interaction between HAdV-5 hexon HVRs and blood coagulation factor X (FX) was demonstrated.^37,38,39,40^ This interaction proved not to be exclusive for HAdV-5 since it also occurs with other HAdV serotypes (members of species HAdV-A, B, C, and D) indicating a conserved trait in HAdV biology,^37^ that may have further relevance in AdV infections as it was suggested for both factor IX (FIX) and FX.^41,42^ At present, there is controversy on whether the interaction between HAdV-5 and FX promotes the innate immune response^43^ or protects HAdV-5 vectors from it.^44^ In this regard, it is relevant to comment that no significant activation of innate immune-relevant primary human mononuclear phagocytes by HAdV-5 loaded with human FX has been observed.^45^

At the liver site, Kupffer cells (KC) and liver sinusoidal endothelial cells act as principal sinks for i.v.-injected HAdV-5 vectors, preventing efficient hepatocyte transduction.^30,46^ The HAdV-5 engulfment by phagocytic KC occurs by several mechanisms including charge-dependent scavenger receptor-A (SR-A),^47,48^ bridging natural IgM antibodies and complement factors.^35^ Nonphagocytic liver sinusoidal endothelial cells are thought to capture HAdV-5 vectors by pinocytosis in a process that may involve scavenger receptor expressed on endothelial cells (SREC-I).^46,48^ In order to attempt liver de-targeting of HAdV-5 vectors, saturation of liver macrophages by pharmacological treatments (*i.e.*, clodronate liposomes) or predosing with a HAdV-5 empty backbone vector has been attempted.^49^ As described earlier, these procedures should be carefully studied in preclinical models as profound damage to liver cells could severely impact overall innate toxicity. Recently, Piccolo *et al.*^50^ showed that KC and liver sinusoidal endothelial cell barriers could be surpassed by helper dependent HAdV-5 (HD HAdV-5) vectors in a mouse model by pretreating the animals with peptides designed to block the scavenger receptors SR-A and SREC-I, increasing hepatocytes transduction and keeping IL-6 levels steady. In addition, a prominent role of the hexon protein in liver entrapment has been illustrated by the reduced liver tropism of HAdV-5 vectors carrying hexon HVRs from either HAdV-6 or HAdV-48 serotypes.^51,52^ As such, research is progressing to find alternative and safer means to de-target HAdV-5 vectors from the liver.

### HAdV preexisting host immunity

Immunological host memory determines the third major issue encountered with HAdV-5 vectors as at early adulthood a large percentage of the humans worldwide carry potent neutralizing antibodies (nAbs) against HAdV-5 and many other HAdV serotypes. Circulating anti-HAdV-5 antibodies have been shown to significantly dampen the ability of HAdV-5 vectors to transfer the gene of interest to the target tissue.^53,54^ Although geographically dependent, anti-HAdV-5 nAbs prevalence have been reported to be over 50% worldwide and even higher in sub-Saharan regions, which is an important region for many AdV-based vaccine strategies including efforts to develop vaccines against human immunodeficiency virus (HIV), *Plasmodium falciparum* (malaria) and *Mycobacterium tuberculosis* (TB).^55,56^ Moreover, high anti-HAdV-5 nAbs titres have been found in human individuals worldwide.^56^ In-depth research demonstrated that the majority of nAbs are targeted to the hexon HVR protein sequences and to a much lesser extent to the fiber knob protein domains.^57^ As a consequence, swapping the HAdV-5 HVRs with HVRs selected from a different AdV serotype suffices to bypass HAdV-5 vector neutralization *in vivo*.^58^ Of note, this neutralization bypass strategy was not achieved when the HAdV-5 fiber knob domain was swapped using a knob domain from a different AdV serotype.^59^ However, the role of anti-fiber nAbs in vector neutralization needs to be further researched as studies to date were performed with animals pre-immunized only once with the AdV vector towards which the acquired immunity was to be overcome.^60^ It has been demonstrated that anti-fiber nAbs are more abundant after two or more immunizations which may better resemble what is actually encountered in nature.^61^ Furthermore, a prominent role in intracellular trafficking has been assigned to the HAdV-5 fiber protein,^62^ a process that has been recently related to the enhancement of cellular antiviral innate immune responses.^63^ Thus, chimeric HAdV-5 vectors with swapped hexon HVRs and fiber could be considered optimal and this strategy warrants further research.^57^

Next to the detrimental effect on gene transfer efficiency of nAbs against HAdV-5 vectors, several studies have demonstrated a widespread existence of HAdVs cross-reactive T cells epitopes.^64,65,66^ Their presence within a majority of the human population, their demonstrated effector and memory poly-functionality, and cross-reactivity among serotypes emphasize their significant role in PEI.^67^ Again, the hexon protein is a major immunological target as it contains the most potent epitopes identified to date,^68,69,70^ although the E2b encoded viral DNA polymerase is also abundantly recognized by cytotoxic T cells at high frequency.^71,72^ Notably, cytotoxic T cell responses have been described to be conserved between diverse HAdV serotypes but also to a certain extent among AdVs isolated from hosts other than humans.^67,73^

Based on the challenges with HAdV-5 vectors described above, strategies to circumvent the observed limitations are being actively researched and include: (i) temporarily altering the host immune system in an attempt to dampen the anti-HAdV-5 immune response, (ii) change the vectors' genomic design, and (iii) modify or shield the HAdV-5 vector capsids.^74^ With regard to strategies that dampen the host immune response, suppression of the host immune system before HAdV-5 vector delivery has been attempted in mouse and nonhuman primate models.^75,76^ Although these strategies achieved some success, they are inherently risky given the fact that eligible patients for gene therapy approaches likely should not be exposed to immune suppressive agents.

With respect to the second strategy, stripping the HAdV-5 vector genome of viral genetic sequences permitted the production of less immunologically visible vectors with larger cloning capacities. For instance, HAdV-5 vectors further deleted of the viral DNA polymerase gene (E2b), and the so-called gutless or helper-dependent vectors, that lack all viral genes and can fit in up to 36 kb of exogenous DNA, have proved advantageous in comparison to E1/E3 deleted HAdV-5 antecedents. Yet, the fundamental role of the capsid itself was shown when HD HAdV-5 recalled early innate immune responses,^77^ and their systemic delivery to baboons resulted in inflammatory shock.^78^ As described earlier, insertion of the viral E3-region back into an E1/E3 deleted HAdV-5 significantly diminished the immune response against the HAdV-5 vector and resulted in prolonged *in vivo* transgene expression.^23^ Similarly, a significant reduction in the anti-HAdV-5 vector innate immune response was accomplished by insertion of the human complement inhibitor decaying-accelerating factor into the HAdV-5 capsid.^79^

The third strategy, *i.e.*, capsid shielding, has been researched for both reduction of hexon HVR-antigen exposure as well as for HAdV-5 vector tropism retargeting.^80,81,82^ However, unless fused to the amino terminus of pIX, the insertional size of shielding moieties is limited, and this approach has demonstrated to severely impact the manufacturability of such “shielded” and/or “re-targeted” vectors given the low yield of replication-deficient HAdV-5 vectors obtained.^83^ Another strategy to shield the antigenic HAdV-5 vector sites is being investigated whereby coating of HAdV-5 capsids using diverse materials, like polymers and lipidic envelopes is attempted.^84^ Less toxic profiles and increased half-life of the vectors in blood were described on i.v. delivery of HAdV-5 vectors shielded with poly[N-(hydroxypropyl)methacrylamide] or polyethylene glycol.^85,86^ Circumvention of HAdV-5 PEI in experimental mouse and nonhuman primate models using this strategy was also described.^87^ Given that polymers interaction with capsid components is noncovalent and unspecific, efforts to determine a more controlled conjugation have been researched, like using the FX protein as a PEGylation adapter^88^ or insertion of a biotin-containing tag in the HVR5-loop of the HAdV-5 hexon protein.^89^ However, these studies also clearly demonstrated that transgene expression was negatively affected by the coat and therefore most recent advances focus on a polymer formulation that can be lost upon vector entry.^90^

Due to the challenges described above with HAdV-5 vectors and the overwhelming evidence that HAdV-5 innate toxicity and PEI are inextricably linked to the capsids protein composition, the research community is actively seeking strategies to alter or exchange HAdV-5 capsid proteins from those of other serotypes and explore the use of vectors based on other AdVs from either human or nonhuman origin.

## Alternatives to HADV-5 Vectors

### HAdV-5–based capsid chimeras

Two decades of intense research have resulted in a thorough understanding of adenovirus biology, cell propagation requirements, genome engineering, and a wealth of basic tools to facilitate the construction of HAdV-5 capsid chimeric vectors. Initially, efforts to pseudotype HAdV-5 focused on the fiber protein to change tropism.^91,92,93,94^ Indeed, fiber-pseudotyped HAdV-5 was demonstrated to alter *in vitro* transduction profiles and for instance create HAdV-5 vectors capable of infecting cell types with low level or no CAR.^95^ For instance, *ex vivo* transduction of human airway epithelium was significantly improved with HAdV-5 vectors pseudotyped with the fiber protein derived from HAdV-35 (HAdV-5F35).^96^ This vector, further engineered to carry the HAdV-35–derived penton base, also proved highly capable of transducing human primary vascular tissue.^97^ Likewise, *in vivo* transduction of muscle cells was significantly improved upon intramuscular injection of a chimeric HD HAdV-5F3 as compared to the standard HAdV-5 vector.^98^ However, despite a clear re-targeting of such vectors *in vitro*, *ex vivo*, and upon local delivery *in vivo*, upon i.v. injection of HAdV-5-based vector mutants, it was clearly demonstrated that other determinants were influencing the *in vivo* tropism.^99,100^

As described earlier, identifying the prominent role of the hexon protein HVR domains in anti-HAdV-5 vector responses and liver targeting led to the successful construction of HAdV-5 vectors with HVR domains derived from less prevalent HAdV serotypes.^58,101^ Here, the identification of a single suppressor mutation in the hexon sequence that allowed for HAdV-5 vector HVR chimeras to be manufactured with near wild-type vector yields has fueled the generation of HVR chimeric vectors.^102^ At present, a vaccine candidate against HIV, based on a HAdV-5 vector carrying the HVR domains from HAdV-48, has been shown to be both safe and immunogenic.^103^ The i.v. delivery of this HAdV-5HVR48 chimeric vector in mice induced high inflammatory cytokine levels that subsequently drove hepatic injury, an effect not observed when the vector was delivered in the muscle.^104^ Data obtained thus far warrant the further development of capsid chimeric HAdV-5 vectors and owing to two recent molecular biology breakthroughs, it can be foreseen that many chimeric vectors will be pushed forward into the clinical product pipeline. These breakthroughs include a high-throughput system for the production of capsid chimeric vectors based on recombination-mediated genetic modification of bacterial artificial chromosomes,^105^ and the demonstration of AdV genome editing by the CRISPR-Cas9 system.^106^ Although promising, capsid exchange strategies significantly impacted folding and assembly of nascent particles giving rise to poor functional titers or complete failure to rescue viable recombinants.^105^ It is therefore that the research community also started to exploit the natural diversity within the *Adenoviridae* family. This has resulted in a number of novel vectors that have been and are being built from AdVs of different serotypes of human and nonhuman origin. Encouragingly, the first data on the safety and efficacy profiles of some of these vectors, including serotypes isolated from either human or nonhuman tissues, have been described^107,108^ (**Figure 1**).

*Alternative human AdV vectors.* Research on HAdV serotypes other than HAdV-5 ignited when the limitations of HAdV-5 repetitive *in vivo* gene transfer protocols and prime-boost vaccination strategies with the same backbone were observed.^109^ In pursue of vectors that would not cross-react, the first non-HAdV-C serotype to be constructed was E1a-deleted HAdV-7 (HAdV-B),^110^ which was successfully produced in a HEK293 cell line stably expressing the HAdV-5 derived E4-ORF6 protein.^111^ Since then, several other members of HAdV-B species were vectorized owing to their tropism profile and low seroprevalence in the human population.^112^ These include vectors based on HAdV-3, -11, -35, and -50 (refs. 107,113,114,115). Of these, HAdV-3 and HAdV-35 have been tested in human subjects as oncolytic vector (replication-competent) and as candidate malaria vaccine expressing *Plasmodium falciparum* circumsporozoite surface antigen (replication-deficient), respectively.^116,117^ Further development of HAdV-B vectors included the engineering of a HAdV-35 providing high transgene expression of two products,^118^ and an improved oncolytic vector based on serotypes 3 and 11 (ColoAd1) obtained by directed evolution.^119^ The ColoAd1 vector demonstrated higher potency and selectivity than the clinically tested ONYX-015 *in vitro* and *in vivo* in a xenograft mouse model.^119^ Importantly, the ColoAd1 vector demonstrated an acceptable biological activity threshold in whole human blood in contrast to the HAdV-5 vector.^120^

A major technological discovery facilitating the rapid construction and production of vectors other than HAdV-5 was the introduction of the HAdV-5–derived ORF6 protein into the viral backbone of the selected serotype. This finding allowed efficient production of non-HAdV-5 replication-deficient vectors (derived either from human or nonhuman tissues) using existing HAdV-5 E1-complementing cells such as HEK293 and PER.C6.^121^ Whereas, HAdV-28 was produced still in the HEK293 ORF6-expressing cells,^122^ the research community rapidly adapted the novel technology resulting in the development of vectors of HAdV-D serotypes 26, 48, and 49.^107,123,124^ Subsequent studies with the novel vectors suggested that they may bind the cellular CD46 protein instead of CAR and that HAdV-26, HAdV-48, as well as HAdV-35 (HAdV-B) induce significantly higher innate immune responses as compared to HAdV-5 in rhesus macaques, a finding that further fueled their development as vaccine vectors.^125^ To date, the HAdV-35 and HAdV-26 have been tested in several phase 1 clinical trials as candidate vaccine component against *Mycobacterium tuberculosis*, *Plasmodium falciparum*, HIV, and Ebola.^126,127,128,129,130^ In healthy adults and toddlers, both vectors have demonstrated excellent safety profiles and their ability to elicit efficient T- and B-cell responses specific against the inserted antigens. As a consequence, a HAdV-26–based anti-HIV vaccine candidate carrying a mosaic HIV envelope antigen that successfully passed phase 1 clinical studies^129,131^ has recently been moved forward to phase 2 trials. The same HAdV-26 vector is also utilized in an experimental two-component vaccine against the highly-lethal Ebola virus Zaire strain that caused the West Africa epidemic in 2014 (ref. 132). As described above, a replication-deficient HAdV-28 vector has also been developed and was shown not to utilize the CD46 receptor. The HAdV-28 vector outperformed the HAdV-35 vector at both moderate and high doses and proved comparable to the HAdV-5 vector in its ability to elicit specific potent T-cell responses to a surrogate influenza virus antigen in mice.^122^ The application of replication-deficient HAdV-48 and HAdV-35 as vaccine vectors needs careful further studying given the observation that they trigger host IFN-α responses which may exert a negative impact on their capability to elicit potent host immune responses.^133^ Yet another vector under study is based on HAdV-49, which utilizes CD46 as primary cellular receptor. *In vivo* experiments demonstrated that the HAdV-49 vector did not trigger a potent CD8^+^ T-lymphocyte–specific response when compared to a HAdV-5 carrying the same SIV Gag antigen. Nonetheless, the HAdV-49 vector clearly remained immunogenic in animals that were preimmunized with a HAdV-5 vector in an attempt to mimic the situation in human individuals.^124^ Furthermore, the HAdV-49 vector, aside from its vaccine vector potential, shows promise as cardiovascular gene therapy vector as it has been shown to transduce primary vascular tissues *in vitro* and *ex vivo* with remarkable efficiency.^134^

Finally, the potential of two other vectors derived from human species HAdV-E (HAdV-4) and HAdV-F (HAdV-41) as vaccine vectors is also being explored. Based on the excellent safety track record of the wild-type HAdV-4 vaccine (given orally in combination with wild-type HAdV-7 as vaccine to US military personnel), the HAdV-4 vaccine vector has the unique feature of being replication-competent with its foreign transgenes cloned into the E3 region instead of the commonly used E1 region.^135,136^ Upon successfully reaching preclinical set points, the HAdV-4 replication-competent vector expressing the influenza H5N1 hemagglutinin protein was tested as an oral vaccine in a phase 1 clinical study in combination with an inactivated H5N1 booster vaccine.^137^ With regard to HAdV-41, it is known that this virus displays two fiber proteins and its penton-base lacks the RGD motif,^138^ which are deemed important characteristics that link this virus to subclinical disease in the human gastrointestinal tract. Given these properties, even though a relatively high seroprevalence had been reported (40–50%),^139,140^ a HAdV-41 vector was constructed. Thus far, the HAdV-41 vector has been shown to enhance intestinal immunity on its own as well as in prime-boost regimes with a HAdV-5 vector carrying the HIV-Env antigen.^141^

The substantial research efforts in recent years in understanding human mastadenovirus biology (clinical and subclinical profile, tropism, seroprevalence, etc.) have exhausted the number of vectors that are potentially capable of circumventing the challenges observed with HAdV-5. As such, the research community has turned its attention to developing vectors from AdVs derived from tissues of nonhuman origin, and results of these scientific efforts will be discussed next.

*Adenoviral vectors derived from nonhuman tissues extracted AdVs.* Nonhuman AdV (NH AdV) vectorization dates back to the 1990s, and many mammalian and avian vectors have since been tested in their homologous hosts^142^ (**Figure 1**). For instance, bovine AdV-3 (BAdV-3), porcine AdV-3 (PAdV-3) and -5, canine AdV-2 (CAdV-2), fowl AdV-1 (FAdV-1), -8, -9, and -10 have been modified to respectively express homologous host-relevant antigens in an attempt to build affordable and effective vaccines.^143,144,145,146,147,148^ In spite of this research effort, none of these approaches have led to the market introduction of any recombinant AdV-based veterinary vaccines. Currently, the only promising such vaccine candidate is a replication-deficient HAdV-5 vector expressing the relevant genes of the foot and mouth disease virus.^149^

The application of NH AdV vectors in human subjects has received strong impetus as many NH AdV abortively infect human cells and do not cross-react with HAdVs.^150^ However, their propagation on existing cell platforms and purification demands might complicate their product development trajectory. Also, it has been described that such serotypes can potentially still share cytotoxic T cell epitopes with HAdV-5 or other HAdV vectors.^66,67,73^ The development process of the canine derived AdV-2 vector (CAdV-2) illustrates the challenges that can be met when developing a nonhuman serotype into a vector that is prone to undergo clinical testing.^151^ Here, the ability of the CAdV-2 vector to transduce neurons, affording durable transgene expression *in vivo*, demonstrated its potential as product to target neurological disorders.^152,153^ Fundamental in vector development has been the development of a good manufacturing procedure-compliant CAdV-2 manufacturing process.^154,155^ Besides, high quality helper-dependent CAdV-2 vectors are under development too.^156^ Such advancement is of striking importance as leaky viral gene expression in cells transduced with AdV vectors bears the risk of the development of broad T-cell responses. In the case of vaccination vectors, this may shadow the specific response against the vaccination antigen, whereas regarding a gene therapy vector application, this phenomenon could involve a reduced expression of the therapeutic product in terms of potency and duration. The development of HD vectors derived from novel serotypes is, generally, problematic because the corresponding packaging signals have not been systematically mapped. In this respect, the work with CAdV-2 HD vector development makes this serotype a flagship for the field.

The success with CAdV-2 renewed the interest in bovine, porcine, and murine AdV vectors (genus *Mastadenovirus*; **Figure 1**), although most of these programs are at a very early developmental stage. It will be very exciting to see these vectors developed given the observation that no cross-reactivity with anti-HAdV nAbs has been observed and the data indicating these vectors can efficiently transduce human cells.^157,158,159,160^ At present, their E1-complementation has been described,^161,162,163^ as well as E3 transgene replacement.^164,165,166^ They respectively rely on sialic acids, integrins, and heparin sulphate proteoglycans for *in vitro* cell entry.^158,167,168^ Both BAdV-3 and PAdV-3 preclinical data indicate the induction of potent innate immune responses which in the absence of cross-reactivity to HAdV-5 make these vectors potentially potent as antigen carriers in vaccine development.^169,170^ Again, further research is required before a conclusion can be obtained as to their vaccine vector utilization as it was also shown for BAdV-3 vector for instance that despite it bypassed HAdV-5 immunity in mouse *in vivo* models, still damaged the liver.^157^

Simian AdVs (SAdVs) are the closest relatives to HAdVs (**Figure 1**), and as a consequence, replication-deficient SAdVs can be manufactured efficiently in existing mammalian cells expressing HAdV-5 E1.^171^ It has been described that some SAdVs can utilize CAR,^172^ but certainly many serotypes are expected to utilize cellular receptors other than CAR. In addition, it has been described that SAdVs differ markedly from HAdV-5 in their antigenic determinants (HVRs and fiber protein domains), and much lower seroprevalences in human individuals worldwide have been reported.^55,173,174^ First-generation SAdV vectors were based on SAdV-25 (chimpanzee, C68), SAdV-22 (Pan 5), SAdV-23 (Pan 6), and SAdV-24 (Pan 7).^175,176^ Given the promising preclincial results with these vectors, characterization of hundreds of novel chimpanzee AdV (“ChAd”) isolates was undertaken and promising candidates, *i.e.*, low seroprevalence in the human population and lack of cross-neutralization with HAdVs, are being selected for further vector development.^177^ Recently, three rhesus monkey-derived AdVs related to *Human mastadenovirus G* were isolated, vectorized, and characterized for their immunogenic properties.^108^ These vectors outperformed an existing ChAd vector as candidate HIV vaccine vectors in a nonhuman primate SHIV challenge study (Dan Barouch, personal communication). To date, at least three vectors based on ChAds have reached clinical trials: strains ChAd3 and ChAd63, respectively, engineered to express hepatitis C virus and *Plasmodium falciparum* antigens,^178,179^ and a chimpanzee AdV strain Y25 construct (vector ChAdOx1) carrying influenza virus derived nucleoprotein and matrix protein antigens.^180^ Like for the HAdV-26 vector described earlier, also a ChAd3-derived vector that expresses an Ebola virus glycoprotein was shown to afford durable protection against lethal challenge in macaques.^181^ Notably, data obtained to date demonstrate that SAdV-derived vectors are capable of triggering potent antigen-specific CD8^+^ T cell responses and thus provide an exciting novel vaccine technology platform.

The aforementioned SAdVs are all members of genus *Mastadenovirus*, hence cell lines, genome manipulation strategies, and test assays could all be easily adapted from the HAdV-5 vectors toolbox and know-how. More “exotic” NH AdVs from other genera lack that benefit but still represent interesting vectors and as such warrant vector development efforts. For instance, fowl AdVs (FAdVs) from the genus *Aviadenovirus* carry an average ~10 kb larger viral DNA genomes as compared to mastadenoviruses and as such could presumably have larger packaging capacity.^1^ Furthermore, FAdVs display two fibers protruding from the same vertex, which in FAdV-1 (FAdV-A) and FAdV-C serotypes FAdV-4 and -10 are encoded by two genes giving rise to distinct long and short fibers that could offer cell-targeting advantages.^182^ Four FAdV serotypes have been vectorized and successfully propagated in avian cells: FAdV-1, -8, -9, and -10.^148,183,184,185^ FAdV-1 (CELO, Chicken Embryo Lethal Orphan) and FAdV-9 (FAdV-D) have been further studied as they abortively infect human cells while yielding high transgene expression unaffected by PEI.^183,186^ Based on these findings, CELO vectors have been constructed to express IL-2, HSV-1 tyrosine kinase, or p53 and have demonstrated long-term gene expression in preclinical models.^187,188,189^

The prototype member of the genus *Atadenovirus*, ovine AdV-7 (OAdV-7), has also been vectorized.^190^ The viral genome of OAdV-7 is A+T-rich and lacks a distinguishable E1 region.^191^ OAdV-7 vectors have the capacity to efficiently deliver foreign transgenes *in vitro* and *in vivo* through abortive infection of a variety of nonovine cells.^192^ Furthermore, OAdV-7–based vectors were shown to overcome anti-HAdV PEI *in vivo*, where liver sequestering is not a dominant biological landmark.^193,194^ These preclinical characteristics supported the further development of OAdV-7 as oncolytic and vaccine vector and to ensure clinical-grade production, a good manufacturing procedure-compliant ovine packaging cell line has been developed.^195^ The preclinical data package, obtained with the OAdV-7 vector further includes several preclinical studies that demonstrate significant induction of antitumor immunity and tumor mass reduction in mice.^196,197^ As vaccine vector, OAdV-7 carrying the NS3 antigen derived from hepatitis C virus elicited a strong T-cell response in mice independent of anti-HAdV-5 PEI. Its performance in prime-boost regimes with recombinant fowlpox virus^198^ and MVA is currently being explored.^199^

*Discovery of novel AdV types.* The promising results thus far obtained with NH AdVs have fuelled the appetite of the research community to isolate novel adenoviruses, and this has triggered substantial discovery programs. Although currently the number of known AdV types in a given host is still the largest for HAdVs, more and more new animal AdVs are rapidly being discovered.^200,201^ Given the variety observed in known adenovirus hosts, the potential of discovering novel AdVs is very high.

In order to classify newly isolated strains, researchers traditionally serotyped them by means of serum neutralization tests.^202^ However, with the rapidly growing number of types, these tests became tedious and time consuming, and they require reliable prototype virus strains and hyperimmune serum collections. For instance, to appropriately serotype a FAdV isolate, 12 standard reference antiserums are needed. Furthermore, to determine whether a novel FAdV serotype has been isolated, the 12 reference FAdV strains are also needed as a serotype is defined as “one that either exhibits no cross-reaction with others or shows a homologous:heterologous titer ratio greater than 16 (in both directions).”^1^ Rapid advances in molecular DNA techniques facilitated the discovery of novel AdVs. For instance, restriction endonuclease analysis of the viral genome was found to be appropriate for differentiating numerous “genotypes” among the isolated HAdV strains.^203,204^ Also, different DNA hybridization techniques have been successfully applied.^205^ However, such techniques became quickly outdated when PCR technology appeared. To date, several PCR systems to detect AdVs have been described, some of which yield positive results only for the specific AdVs they had been designed for.^206,207^ However, the nested PCR that targets the most conserved part of the viral DNA-dependent DNA polymerase gene has proven to be extremely useful even in the recognition of previously unknown AdVs.^208^

As the cost of DNA sequencing decreases owing to rapid technology improvements, full genome sequence analysis has become a routine technique allowing characterization of microorganisms at their full genome level. Clearly, such technologies have taken the speed at which novel adenoviruses can be discovered, typed, characterized, and vectorized to an unprecedented level. These technologies and recent advances in deep sequencing, which can yield useful results even if a pathogen is present in a very small quantity in a clinical sample,^209^ will undoubtedly aid in understanding the complexity of the adenovirus family, its evolution, and how to develop novel vectors that can be used to battle human diseases.

## Concluding Remarks

The *Adenoviridae* represents a large and varied family with members present in representatives of most species looked at today be it mammalian, avian, or reptilian, and we may have only started to understand its complexity. The wealth of results generated, mainly on HAdV-5 as model, significantly contributed to master their biology and develop tools to enable vector production, purification, and genetic engineering for medicinal purposes. Building on such knowledge, nowadays AdV vectors are the most represented in ongoing clinical trials and a rapidly expanding portfolio of vectors for biomedical application in gene therapy, oncotherapy, and vaccination is being developed. Understanding their weaknesses and strengths allows for the rational reengineering of AdVs to ensure safe and efficient delivery of foreign DNA to target tissues and cells with the purpose to trigger potent immune responses or long-term gene expression. With first products based on AdVs already approved in man, it can be envisioned that in the foreseeable future, a generation of novel safe and potent therapeutic and preventive medicines based on them will be available to battle human disease.
